# Tissue-specific biological aging predicts progression in prostate cancer and acute myeloid leukemia

**DOI:** 10.3389/fonc.2023.1222168

**Published:** 2023-09-06

**Authors:** Anitha Ramakrishnan, Indrani Datta, Sukanya Panja, Harmony Patel, Yingci Liu, Michael W. Craige, Cassandra Chu, Giselle Jean-Marie, Abdur-Rahman Oladoja, Isaac Kim, Antonina Mitrofanova

**Affiliations:** ^1^ Department of Biomedical and Health Informatics, School of Health Professions, Rutgers, The State University of New Jersey, Newark, NJ, United States; ^2^ Department of Health Informatics and Information Management, College of Applied and Natural Sciences, Louisiana Tech University, Ruston, LA, United States; ^3^ New Jersey Medical School, Rutgers, The State University of New Jersey, Newark, NJ, United States; ^4^ Rutgers Youth Enjoy Science Program, Rutgers Cancer Institute of New Jersey, New Brunswick, NJ, United States; ^5^ Department of Urology, Yale School of Medicine, New Haven, CT, United States; ^6^ Rutgers Cancer Institute of New Jersey, Rutgers, The State University of New Jersey, New Brunswick, NJ, United States

**Keywords:** genome-wide analysis, biological aging, biomarkers, cancer progression, precision medicine

## Abstract

**Introduction:**

Chronological aging is a well-recognized diagnostic and prognostic factor in multiple cancer types, yet the role of biological aging in manifesting cancer progression has not been fully explored yet.

**Methods:**

Given the central role of chronological aging in prostate cancer and AML incidence, here we investigate a tissue-specific role of biological aging in prostate cancer and AML progression. We have employed Cox proportional hazards modeling to associate biological aging genes with cancer progression for patients from specific chronological aging groups and for patients with differences in initial cancer aggressiveness.

**Results:**

Our prostate cancer-specific investigations nominated four biological aging genes (CD44, GADD45B, STAT3, GFAP) significantly associated with time to disease progression in prostate cancer in Taylor et al. patient cohort. Stratified survival analysis on Taylor dataset and validation on an independent TCGA and DKFZ PRAD patient cohorts demonstrated ability of these genes to predict prostate cancer progression, especially for patients with higher Gleason score and for patients younger than 60 years of age. We have further tested the generalizability of our approach and applied it to acute myeloid leukemia (AML). Our analysis nominated three AML-specific biological aging genes (CDC42EP2, CDC42, ALOX15B) significantly associated with time to AML overall survival, especially for patients with favorable cytogenetic risk score and for patients older than 56 years of age.

**Discussion:**

Comparison of the identified PC and AML markers to genes selected at random and to known markers of progression demonstrated robustness of our results and nominated the identified biological aging genes as valuable markers of prostate cancer and AML progression, opening new avenues for personalized therapeutic management and potential novel treatment investigations.

## 1 Introduction

Chronological aging is a widely recognized diagnostic and prognostic factor across a variety of diseases, including oncogenic and nononcogenic malignancies. In recent years, biological age has received a lot of attention in attempts to slow down the onset of aging and reverse age-related deterioration (1–4). As opposed to the chronological age of how long a person has existed, biological age is defined based on how old a person’s cells are, reflecting one’s functionality and onset of diseases related to old age. The role of biological aging has recently received a lot of attention in Alzheimer’s disease and multiple sclerosis (1–4), but its role in oncology remains a largely unexplored promising new direction with potential for more effective biomarker discovery and novel therapeutic strategies.

Chronological age is a known central player in prostate cancer (PC) incidence, as more than 65% of all prostate cancer cases are diagnosed after the age of 65, with incidences increasing with each year of age. While chronological age is not a prognostic biomarker in prostate cancer (i.e., disease aggressiveness is not correlated with age at diagnosis), the prognostic role of *biological* age in this malignancy has not been investigated to date.

Our state-of-the-art literature investigations uncovered nontissue-specific 52 genes associated with biological aging, including genes from the hippocampus associated with Alzheimer’s disease (1), multiple tissues in association with cognitive health (2), skeletal muscles associated with aging (3), and genes associated with aging and mortality (4).

Among the nontissue-specific 52 genes associated with biological aging (1–4), four genes (CD44, GADD45B, STAT3, and GFAP) showed prostate cancer tissue-specific association with disease progression in the Taylor et al. primary prostate cancer cohort when adjusted for initial Gleason score (which reflects histologic patterns with prognostic significance in PC) and chronological age. Further stratification of this cohort into subgroups of patients with low and high Gleason scores and younger and older age and subsequent validation in TCGA and DKFZ patient cohorts demonstrated that the four prostate cancer-specific biological aging genes are particularly predictive of PC progression in patients with a higher Gleason score (above 3 + 4) and for patients of younger age (≤ 60 years).

Furthermore, we have tested the generalizability of our approach and applied it to acute myeloid leukemia (AML) with overall survival as a clinical endpoint, identifying three genes (CDC42EP2, CDC42, and ALOX15B) that demonstrated association with AML overall survival in the BEAT AML bone marrow patient cohort (5), adjusted for cytogenetic risk (which reflects clinicopathologic features with prognostic significance in AML) and chronological age. Further stratification of this cohort into subgroups of patients with favorable and unfavorable cytogenetic risk scores and younger and older age and validation in TCGA LAML patient cohort (6) demonstrated that the three AML-specific biological aging genes are particularly predictive of AML overall survival in patients with favorable cytogenetic scores and for patients of older age (> 56).

Finally, comparison to an equally sized set of genes selected at random and to known markers of PC and AML progression, respectively, demonstrated the nonrandom, independent, robust ability of the identified biological aging genes to predict either PC or AML progression, as applicable. Our results indicate that the identified biological aging markers present an independent line of evidence for patient stratification and predictive modeling in addition to initial cancer aggressiveness and patient chronological age and thus could be effectively utilized for personalized disease management and therapeutic advice.

## 2 Methods

### 2.1 Biological aging genes

The literature search identified 52 nontissue-specific genes associated with biological aging, including genes from the hippocampus associated with Alzheimer’s disease (1), multiple tissues associated with cognitive health (2), skeletal muscles associated with aging (3), and genes associated with aging and mortality (4) (**Supplementary Table S1**, **Supplementary Materials**).

### 2.2 Methods for prostate cancer analysis

#### 2.2.1 Prostate cancer patient cohorts utilized in this study

PC gene expression patient cohorts utilized for this study included (i) the Taylor et al. (7) cohort, used for model training/discovery; (ii) The Cancer Genome Atlas Prostate Cancer Adenocarcinoma (TCGA PRAD) cohort (6), used for test/validation; and (iii) the Deutsches Krebsforschungszentrum (DKFZ, The German Cancer Research Center) PRAD cohort (8), used for additional test/validation (**Table 1**).

**Table 1:** Clinical characteristics of prostate cancer patient cohorts utilized in this study.

Characteristics	Training cohort (Taylor et al.)	Test cohort 1 (TCGA PRAD)	Test cohort 2 (DKFZ PRAD)
**Number of samples**	138	384	100
**Number of BCR events**	35/138 (25.36%)	49/384 (12.76%)	24/100 (24%)
**Number of PFS events**	NA	74/384 (19.27%)	NA
**Platform**	Affymetrix Human Exon 1.0 ST Array	Illumina HiSeq 2000	Illumina HiSeq 2000
Age			
≤ 60	87	175	100
> 60	51	209	NA
Gleason score			
3 + 3	41	38	11
3 + 4	53	121	57
3 + 5	1	7	0
4 + 3	23	80	18
4 + 4	8	45	1
4 + 5	10	88	13
5 + 3	2	5	0

The Taylor et al. cohort (7) is an MSKCC dataset composed of primary tumor prostatectomy samples (*n* = 138), profiled on the Affymetrix Human Exon 1.0 ST Array, and obtained from the Gene Expression Omnibus (GEO) GSE21032. Gene expression data were normalized using the robust multi-array average (RMA) algorithm for background correction, quantile normalization, and log2-transformation. The clinical endpoint was defined as a time to biochemical recurrence (BCR) or time to the latest follow-up (censored data).

TCGA PRAD cohort (6) is composed of primary tumor prostatectomy samples (*n* = 384), profiled on Illumina HiSeq 2000, with its RNA-sequencing and clinical data obtained from dbGap phs000178 and cBioPortal. TCGA dataset was mapped to hg19 using the STAR aligner (9). Raw counts were normalized, and variance was stabilized using DEseq2 normalization (10). Two clinical endpoints were considered: (i) time to BCR (defined as a rise of prostate-specific antigen in patient’s blood)/latest follow-up (censored data); and (ii) time to progression-free survival (PFS, defined as the progression of cancer, local recurrence, distant metastases, or PC-specific death)/latest follow-up.

The DKFZ PRAD cohort (8) is composed of primary tumor prostatectomy samples (*n* = 100) profiled on Illumina HiSeq 2000, with RNA-sequencing and clinical data obtained from cBioPortal. Gene expression data were obtained from cBioPortal as RPKM values, z-scored relative to all samples. The clinical endpoint was defined as a time to BCR or time to the latest follow-up (censored data).

#### 2.2.2 Identification of PC-specific biological aging genes associated with PC progression

To identify PC-specific biological aging genes associated with prostate cancer progression, we employed Cox proportional hazards analysis using the *coxph* function from the R survival package (11) on the Taylor et al. training cohort. The 52 biological aging genes were used as input variables, and time to BCR was used as a clinical endpoint in the univariable Cox proportional hazards analysis on the Taylor et al. patient cohort, adjusted for Gleason score (combined major plus minor Gleason was used for adjustment purposes) and chronological age variables (adjusted hazard *p*-value of < 0.05 was considered significant, **Supplementary Table S2**, **Supplementary Materials**).

To assess for multicollinearity among the identified (four) PC genes, we employed the variance inflation factor (VIF) analysis using the *vif* function from the R car package (11). The threshold of 5 was utilized to evaluate the presence of multicollinearity (all evaluated genes had values below 1.5).

For multivariable additive Cox proportional hazards modeling, expression levels of four identified genes were utilized as input variables and were adjusted for Gleason score and chronological age (as above). An adjusted Wald test *p*-value of < 0.05 was considered significant.

#### 2.2.3 PC stratified survival analysis

To investigate the predictive ability of the biological aging genes in subsets of patients defined by Gleason scores and chronological age, we employed cohort stratification. For this, the training Taylor et al. cohort was divided into two Gleason score groups: a group consisting of patients with Gleason scores 3 + 3 and 3 + 4, considered least aggressive clinically (*n* = 94), and the group consisting the rest of the patients (*n* IL = 44). For chronological age-stratified analysis, the Taylor et al. cohort was divided into two age groups: a group consisting of patients aged less than equal to a median age (in the Taylor et al. training cohort) of 60 (*n* = 87) and a group consisting of patients aged over 60 (*n* = 51).

Multivariable additive Cox proportional hazards modeling was run utilizing four identified genes as input variables and time to BCR as a clinical endpoint for each stratified group separately.

#### 2.2.4 PC testing/validation analysis

For testing/validation purposes, a multivariable additive Cox proportional hazards model in a stratified setting was performed using TCGA PRAD and DKFZ PRAD patient cohorts. For Gleason stratification (similarly to the training cohort), two subgroups were defined: the group that included patients with Gleason 3 + 3 and 3 + 4 (TCGA *n* = 159, DKFZ *n* = 68) and the group that included the rest of the patients (TCGA *n* = 225, DKFZ *n* = 32). For chronological age stratification, two subgroups were defined as in training: the group that included patients aged ≤ 60 (TCGA *n* = 175, DKFZ *n* = 100) and the group that included patients aged > 60 (TCGA *n* = 209, DKFZ = NA as this dataset only included patients younger than 6 years of age). The Cox proportional hazards model and *p*-values were estimated as above (i.e., during training).

### 2.3 Methods for acute myeloid leukemia analysis

#### 2.3.1 AML patient cohorts utilized in this study

AML gene expression patient cohorts utilized in this study included (i) the BEAT AML cohort (5), used for model training/discovery; and (ii) TCGA AML (also referred to as TCGA LAML) (6), used for model test/validation (**Table 2**).

**Table 2:** Clinical characteristics of AML patient cohorts utilized in this study.

Characteristics		Training cohort (BEAT AML)	Test cohort (TCGA AML)
**Number of *de-novo* cases (bone marrow)**		127	151
**Number of events (overall survival)**		46/127 (36.22%)	97/151 (64.24%)
**Platform**		Illumina HiSeq 2500	Illumina HiSeq2000
Age			
≤ 56		64	76
> 56		63	75
Cytogenetic risk			
Favorable		53	30
Unfavorable		74	121

The BEAT AML (5) dataset is composed of bone marrow samples from *de novo* AML (*n* = 127), profiled on Illumina HiSeq 2500, with its RNA-sequencing and clinical data obtained from dbGap phs001657.v2.p1 and downloaded through the Genomic Data Commons (GDC). BAM files were converted to FASTQ files, which were then mapped to hg38 using the STAR aligner (9). Raw counts were normalized, and variance was stabilized using DEseq2 normalization (10). Overall survival was utilized as a clinical endpoint.

TCGA AML (6) dataset is composed of bone marrow samples from *de novo* AML (*n* = 151), profiled on Illumina HiSeq2000, with its RNA-sequencing and clinical data obtained from TCGA phs000178.v11.p8 and downloaded through the GDC. BAM files were converted to FASTQ files, which were then mapped to hg38 using the STAR aligner (9). Raw counts were normalized, and variance was stabilized using DEseq2 normalization (10). Overall survival was utilized as a clinical endpoint.

#### 2.3.2 Identification of AML-specific biological aging genes associated with AML progression

To identify AML-specific biological aging genes associated with AML progression, we employed Cox proportional hazards analysis using the *coxph* function from the R survival package (11) as above on the BEAT AML training cohort. The 52 biological aging genes were used as input variables, and overall survival was used as a clinical endpoint in the univariable Cox proportional hazards analysis, adjusted for cytogenetic risk score (based on ELN2017 guidelines (12), which utilize cytogenetics and mutation patient profiles and includes favorable, intermediate, and poor risk scores) and chronological age variables (an adjusted hazard *p*-value of< 0.05 was considered significant, **Supplementary Table S3**, **Supplementary Materials**). To ensure an appropriate sample size for each group, we combined intermediate and poor cytogenetic risk score categories into the “unfavorable” category.

Multicollinearity was assessed as above, with all evaluated genes having values below 1.5. For multivariable additive Cox proportional hazards modeling, expression levels of three identified genes were utilized as input variables and were adjusted for cytogenetic score and chronological age (as above). An adjusted Wald test *p*-value of < 0.05 was considered significant.

#### 2.3.3 AML stratified survival analysis

To investigate the predictive ability of the biological aging genes in subsets of patients defined by cytogenetic risk and chronological age, we employed cohort stratification. For this, the training BEAT AML cohort was divided into two cytogenetic risk groups (based on ELN2017 guidelines): consisting of patients with favorable cytogenetic risk (*n* = 53) and unfavorable cytogenetic risk (*n* = 74, which included patients with intermediate and poor cytogenetic risk scores, for sample size purposes). For chronological age-stratified analysis, the BEAT AML cohort was divided into two age groups: patients with a median age of ≤ 56 in the BEAT AML training cohort (*n* = 64) and patients aged > 56 (*n* = 63).

Multivariable additive Cox proportional hazards modeling was run utilizing three identified genes as input variables and overall survival as a clinical endpoint for each stratified group separately.

#### 2.3.4 AML testing/validation analysis

In AML, for testing/validation purposes, a multivariable additive Cox proportional hazards model in a stratified setting was performed using TCGA AML patient cohort. For cytogenetic stratification (similarly to the training cohort), two groups were defined: the group that included patients with favorable cytogenetic risk (*n* = 30) and a group that included patients with unfavorable cytogenetic risk (*n* = 121). For chronological age stratification, two groups were similarly defined: a group that included patients aged ≤ 56 (*n* = 76) and a group that included patients aged > 56 (*n* = 75). The Cox proportional hazards model and *p*-values were estimated as above (i.e., during training).

### 2.4 Comparison to genes selected and random and to known markers of PC and AML progression

For both PC and AML analyses, to compare the predictive ability of identified biological aging genes to the predictive ability of the equally sized (i.e., four or three, respectively) set of genes selected at random, we defined a random model using the Taylor et al. patient cohort (for PC analysis) and BEAT AML cohort (for AML analysis). In this model, four (for PC) or three (for AML) genes were selected at random and subjected (as a group) to multivariable additive Cox proportional hazards model analysis, with a Wald test *p*-value reported. This process was repeated 10,000 times, and a nominal *p*-value for the random model was calculated as the number of times the random model reached or outperformed the *p*-value for the original (non-random) biological aging genes. The tidyverse, survival, and factoextra R packages were utilized for this analysis.

For comparison to known markers of PC and AML progression, respectively, we employed a univariable Cox proportional hazards model with adjustment for covariates such as Gleason score and chronological aging (for PC) and cytogenetic score and chronological aging (for AML).

### 2.5 Statistical considerations

All statistical analysis was performed using R Studio version 4.4.1. The Cox proportional hazards model was utilized for training/discovery and testing/validation purposes, with and without adjustment for clinical variables, as appropriate. The significance of the analysis was estimated using a hazard *p*-value or Wald *p*-value, where appropriate. To investigate pathway membership of the four (for PC) or three (for AML) identified genes, we utilized the DAVID bioinformatics tool (13) to perform pathway enrichment analysis, where the FDR-corrected chi-square test *p*-value of< 0.05 was considered significant. Integration of *p*-values was done using the harmonic mean method, which is well suited for our analysis as it produces significant results when all/majority of the input *p*-values are significant (14).

For two *p*-values *x* and *y*, the harmonic mean is defined as:


$$\text{Harmonic mean}\;\left(x,y\right)=\;\frac{2*x*y}{x+y}$$

For three *p*-values *x* and *y*, the harmonic mean is defined as:


$$\text{Harmonic mean }\left(x,y,z\right)=\;\frac{2*x*y*z}{x*y+y*z+x*z}$$

Integrated *p*-values (from the harmonic mean calculation) were then FDR-corrected, with FDR-corrected integrated *p*-values of< 0.05 considered significant. Patient cohorts for training/discovery and for testing/validation were obtained from the GEO, dbGap, GDC, and cBioPortal public repositories.

## 3 Results

**Figure 1:**
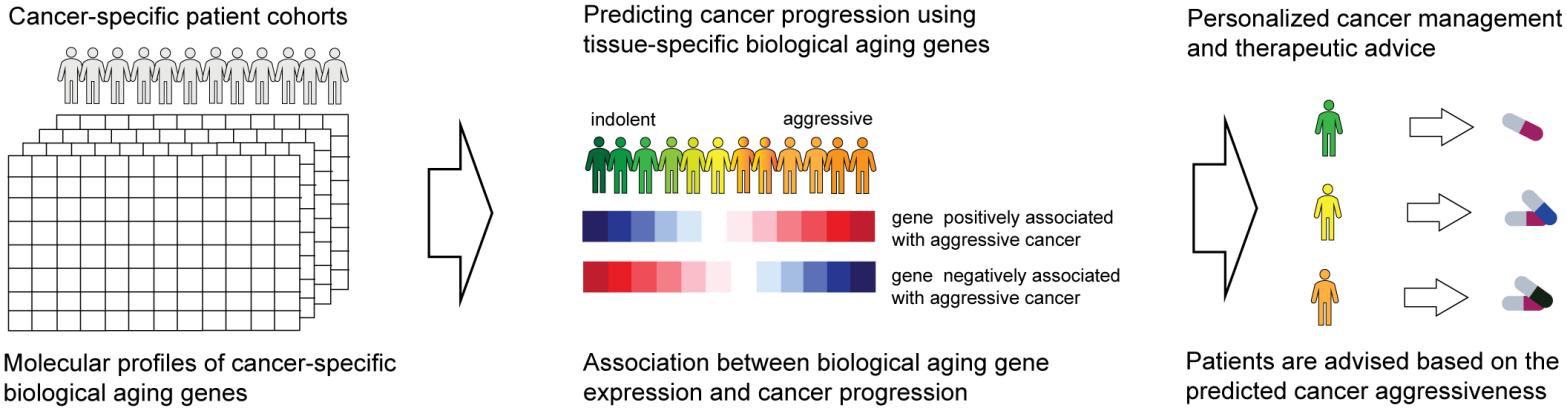
Overall schematics and objectives of our approach. Patient gene expression profiles are examined for the activity of biological aging genes (left). This allows us to assign each patient a score based on the risk of disease progression (middle). The scores are utilized to provide personalized therapeutic advice (right).

The overall objective of our investigations was to evaluate biological aging genes for their ability to predict cancer-specific disease progression across patient cohorts (**Figure 1**, left). The ultimate goal is for each patient to be assigned a disease aggressiveness (i.e., risk) score based on the activity levels of these genes (**Figure 1**, middle). These scores would then be utilized to allow for personalized disease management and therapeutic advice (**Figure 1**, right).

### 3.1 Investigating biological aging genes

To identify nontissue-specific genes associated with biological aging, we have reviewed the current state of the literature and identified 52 genes associated with biological aging, including genes from the hippocampus associated with Alzheimer’s disease (1) (*n* = 28), multiple tissues associated with cognitive health (2) (*n* = 15) and age prediction (15) (*n* = 2), and skeletal muscles associated with aging (3) (*n* = 5), associated with aging and mortality (4) (*n* = 1), and associated with premature aging (16) (*n* = 1) (**Supplementary Table S1**, **Supplementary Material**).

### 3.2 Prostate cancer analysis

#### 3.2.1 Model training/discovery: identifying prostate cancer-specific biological aging genes associated with prostate cancer progression

**Figure 2:**
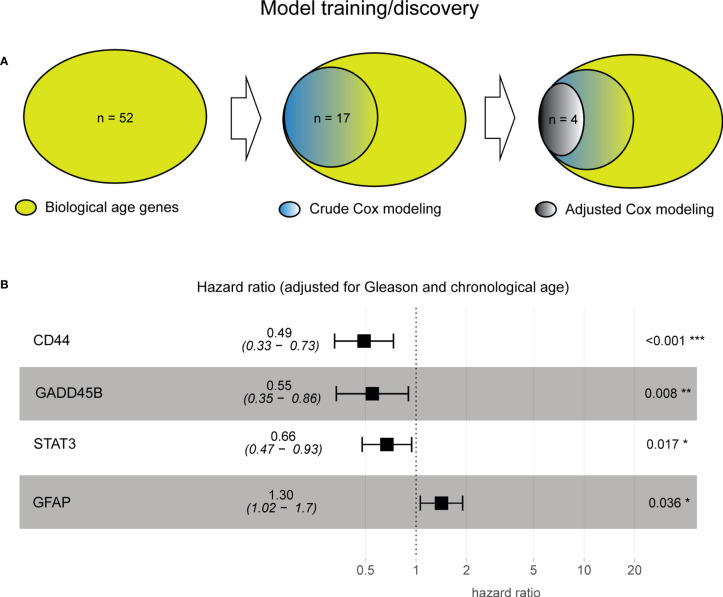
Model training identified four prostate cancer-specific biological aging genes associated with cancer progression. **(A)** Venn diagram from prostate cancer-specific Cox proportional hazards model analysis, ranging from 52 to 17 to four biological aging genes. **(B)** A composite hazards plot, where the analysis for each gene was adjusted for Gleason score and chronological age. Central squares indicate the hazard ratio (HR), and whiskers indicate the HR confidence interval. Hazard *p*-values are indicated (^*^ *p*< 0.05; ^**^ *p*< 0.01; ^***^ *p*< 0.001).

Since the 52 biological aging genes were nontissue-specific, our first objective was to assign their tissue specificity for prostate cancer. For this, we employed the Taylor et al. patient cohort (*n* = 138, see Methods), which comprised primary prostatectomy samples from patients with prostate cancer, with subsequent disease progression monitoring (defined as time to BCR). Taylor et al. dataset was specifically selected for training purposes as it presented a wide chronological age range (37.3–83.0) and contained 25% of patients with BCR failure events, making it an ideal dataset for training purposes. We utilized 52 genes as input variables in the univariable Cox proportional hazards model analysis, with time to BCR or the latest follow-up (if censored) as a clinical endpoint (**Figure 2A**, left). This analysis identified 17 genes with unadjusted Cox proportional hazards *p*-value of< 0.05 (**Figure 2A**, middle) that are significantly associated with prostate cancer progression in a tissue-specific manner. To ensure that our results are not due to the effect of the initial disease aggressiveness (i.e., Gleason score at diagnosis) or the effect of chronological age (at diagnosis), we adjusted our Cox proportional hazards model for these variables and identified four (4) genes that remained significant (adjusted Cox proportional hazards *p*-value of< 0.05) after adjusting for both Gleason score and chronological age (**Figure 2A**, right; **Supplementary Table S2**, **Supplementary Materials**).

Four genes (**Figure 2B**) included those with HR ≤ 1 (negative association with BCR; the higher the gene expression, the poorer the prognosis the patient will have), such as CD44 (Cox proportional hazards model HR = 0.49, *p*-value of< 0.001), GADD45B (Cox proportional hazards model HR = 0.55, *p*-value = 0.008), STAT 3 (Cox proportional hazards model HR = 0.66, *p*-value = 0.017), and HR > 1 (positive association with BCR, the higher the gene expression, the more favorable the prognosis the patient will have) with BCR, such as GFAP (Cox proportional hazards model HR = 1.30, *p*-value = 0.036), demonstrating their significant association with BCR after subtracting the effect of Gleason score and chronological age (**Supplementary Table S2**, **Supplementary Materials**).

Furthermore, to evaluate if the four genes could be utilized as a group for clinical purposes, we first assessed if there exists a multicollinearity among them using VIF analysis (see Methods, no multicollinearity identified), followed by the multivariable additive Cox proportional hazards model analysis (adjusted for Gleason and chronological age, as above), which demonstrated a significant benefit of evaluating the four genes as one additive model (Wald test *p*-value = 2.014−*e*9).

Finally, to evaluate pathway membership for the four identified genes, we employed the DAVID tool for pathway enrichment analysis (13) and identified two significant molecular pathways: the JAK-STAT and the FoxO signaling pathways (FDR-corrected chi-square test *p*-value of< 0.05). The JAK-STAT signaling pathway is involved in a wide range of cellular processes, including cell proliferation, differentiation, and survival (17, 18). The FoxO signaling pathway has been shown to regulate cellular processes such as apoptosis, cell cycle arrest, and DNA damage repair, which are important in maintaining genomic stability and preventing cancer development (19, 20). Both of these pathways have previously been shown to be implicated in biological age-related deterioration and disorders (21–23), and their further investigation might provide valuable insights into the underlying molecular mechanisms involved in prostate cancer biological age-related changes.

#### 3.2.2 Stratified survival analysis

**Figure 3:**
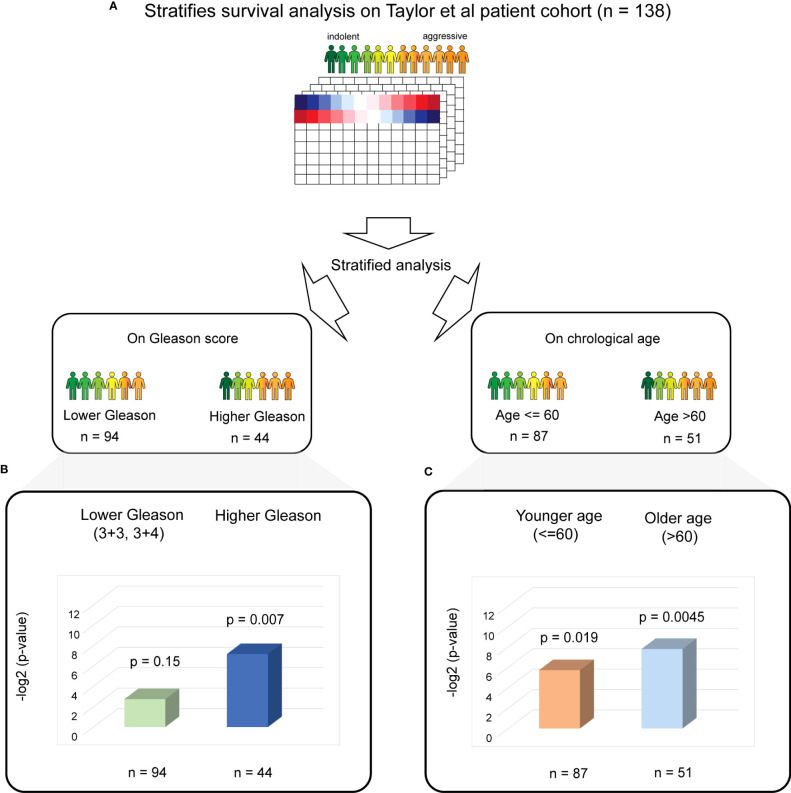
Stratified PC survival analysis identifies the significant predictive ability of four biological aging gene panel in the Taylor et al. cohort. **(A)** The overall strategy for stratified survival analysis in the Taylor et al. patient cohort. Patients are divided into lower and higher Gleason score groups (left) and younger and older chronological age groups (right), with subsequent analysis carried out for each group. **(B)** Cox proportional hazards model analysis for the lower Gleason score patient subgroup (left) and the higher Gleason score patient subgroup (right). Wald test *p*-values and number of patients in each subgroup are indicated. **(C)** Cox proportional hazards model analysis for the younger chronological age patient subgroup (left) and the older chronological age patient subgroup (right). Wald test *p*-values and number of patients in each subgroup are indicated.

To further investigate if the significant association of the four biological aging genes with BCR is attributed to specific patient subgroups (that is driving significance of the association), we have performed stratified Cox proportional hazards analysis on the patient subgroups stratified by the (1) Gleason score, which is a commonly used prognostic factor for prostate cancer, and (2) chronological age, which is a known diagnostic factor for prostate cancer (**Figure 3A**).

For stratified analysis based on the Gleason score, we divided the Taylor et al. patient cohort into two patient subgroups: (i) lower Gleason score (3 + 3 and 3 + 4, commonly utilized in clinic as an indicator of favorable prognosis, *n* = 94) and (ii) a higher Gleason score (higher than 3 + 4, *n* = 44). A multivariable additive Cox proportional hazards model utilizing four identified genes as predictors and time to BCR as response was then performed for each subset separately (**Figure 3B**). This analysis demonstrated a significant association with BCR in a subset of patients with higher Gleason scores (Cox proportional hazards model Wald test *p*-value = 0.007).

For stratified analysis based on chronological age, we divided the Taylor et al. patient cohort into two patient subgroups: (i) younger chronological age (≤ 60, *n* = 87) and older age (> 60, *n* = 51), where 60 years of age is a commonly utilized threshold to estimate prostate cancer risk and also corresponds to the mean and median for the chronological age for this cohort. Multivariable additive Cox proportional hazards model on each chronological age subgroup separately (**Figure 3C**) demonstrated a significant association between the four identified biological aging genes and BCR (Cox proportional hazards model Wald *p*-value = 0.019 for younger chronological age group and *p*-value = 0.0045 for older chronological age group).

#### 3.2.3 Model testing/validation

To validate our findings in independent patient cohorts, we have utilized (i) TCGA PRAD patient cohort (*n* = 384, see **Methods**) and (ii) the DKFZ PRAD patient cohort (*n* = 100, see Methods), both with comparable to the Taylor et al. sample collection protocol (i.e., primary prostatectomy samples) and clinical endpoint (i.e., time to biochemical recurrence).

**Figure 4:**
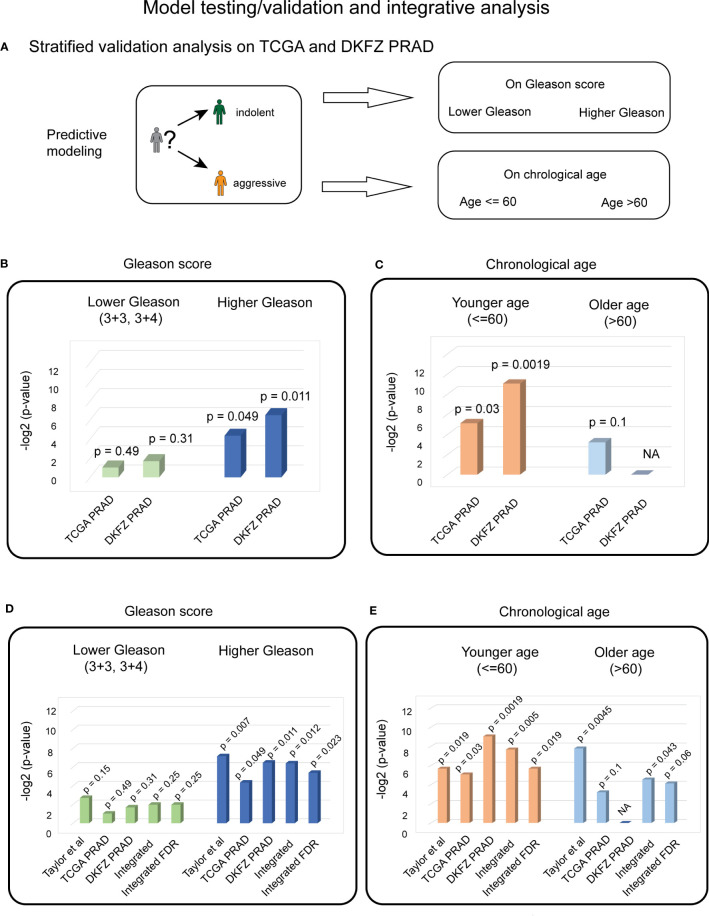
Stratified validation PC analysis indicates the significant predictive ability of four biological aging genes for patients with higher Gleason scores and younger ages. **(A)** Overall strategy for stratified validation analysis in TCGA and DKFZ PRAD test patient cohort. Patients are divided into lower and higher Gleason score groups (top) and younger and older chronological age groups (bottom), with subsequent analysis carried out for each group. **(B)** Cox proportional hazards model analysis for the lower Gleason score patient subgroup (left) and the higher Gleason score patient subgroup (right) in TCGA and DKFZ PRAD cohorts. Wald test *p*-values are indicated. **(C)** Cox proportional hazards model analysis for the younger chronological age patient subgroup (left) and the older chronological age patient subgroup (right) in TCGA and DKFZ PRAD cohorts. Wald test *p*-values are indicated. **(D)** Cox proportional hazards model analysis for the lower Gleason score patient subgroup (left) and the higher Gleason score patient subgroup (right) across Taylor et al., TCGA PRAD, and DKFZ PRAD cohorts. Wald test *p*-values for each dataset and integrated (harmonic mean) *p*-values with and without FDR correction are indicated. **(E)** Cox proportional hazards model analysis for the younger chronological age patient subgroup (left) and the older chronological age patient subgroup (right) across Taylor et al., TCGA PRAD, and DKFZ PRAD cohorts. Wald test *p*-values for each dataset and integrated (harmonic mean) *p*-values with and without FDR correction are indicated.

For testing the predictive ability of the identified four biological aging genes in TCGA PRAD dataset, it was stratified on Gleason score, similarly to training, into (i) Gleason scores 3 + 3 and 3 + 4 (*n* = 159); and (ii) Gleason scores higher than 3 + 4 (*n* = 225) (**Figure 4A**). Similarly to the Taylor et al. dataset, chronological age stratification on TCGA PRAD cohort defined (i) a group characterized by age ≤ 60 (*n* = 175) and (ii) a group characterized by age > 60 (*n* = 209) (**Figure 4A**).

Stratified analysis on the Gleason score demonstrated a significant association of the four biological aging genes to BCR in the higher Gleason score group in TCGA PRAD cohort (Cox proportional hazards model Wald test *p*-value = 0.049, **Figure 4B**). Stratified analysis on chronological age, demonstrated a significant association of four biological aging gene panel with BCR in the younger age (≤ 60) in TCGA PRAD dataset (Cox proportional hazards model Wald test *p*-value = 0.029, **Figure 4C**).

To confirm these findings in another PC patient cohort, we utilized the DKFZ PRAD dataset that was similarly stratified on Gleason score into (i) groups with Gleason scores 3 + 3 and 3 + 4 (*n* = 68); and (ii) groups with Gleason scores higher than 3 + 4 (*n* = 32) (**Figure 4A**). The DKFZ PRAD cohort only included patients aged below or at 60 (*n* = 100). The stratified analysis demonstrated significant association of the four biological aging genes to BCR in the higher Gleason score group (Cox proportional hazards model Wald test *p*-value = 0.011, **Figure 4B**) and in the younger age group (≤ 60) (Cox proportional hazards model Wald test *p*-value = 0.0019, **Figure 4C**), confirming results from TCGA PRAD dataset.

To make final conclusions, we have further integrated our findings from all three cohorts (Taylor et al., TCGA PRAD, and DKFZ PRAD) using FDR-corrected harmonic mean method (**Figures 4D, E**; **Methods**), which nominated a significant association of the identified genes with BCR in the PC patient group with a higher Gleason score (FDR-corrected integrated *p*-value = 0.023) (**Figure 4D**) and younger age (≤ 60) subgroup (FDR-corrected integrated *p*-value = 0.019) (**Figure 4E**), indicating that the four biological aging genes could be utilized as markers to predict BCR in patients with a higher Gleason score and with a younger age, potentially allowing for personalized disease management and therapeutic planning in PC patients.

Furthermore, we expanded our investigations in TCGA dataset to evaluate if our four genes could predict PFS (defined as the progression of cancer, local recurrence, distant metastases, or PC-specific death). Our analysis demonstrated the ability of the four identified genes to predict PFS in the whole TCGA PRAD cohort in an additive Cox proportional hazards model (Wald test *p*-value = 0.034). Even though we observed a significant association with PFS, we anticipate that different biological aging genes might be involved in processes that govern BCR, PFS, or OS, requiring further investigations.

### 3.3 AML analysis

**Figure 5:**
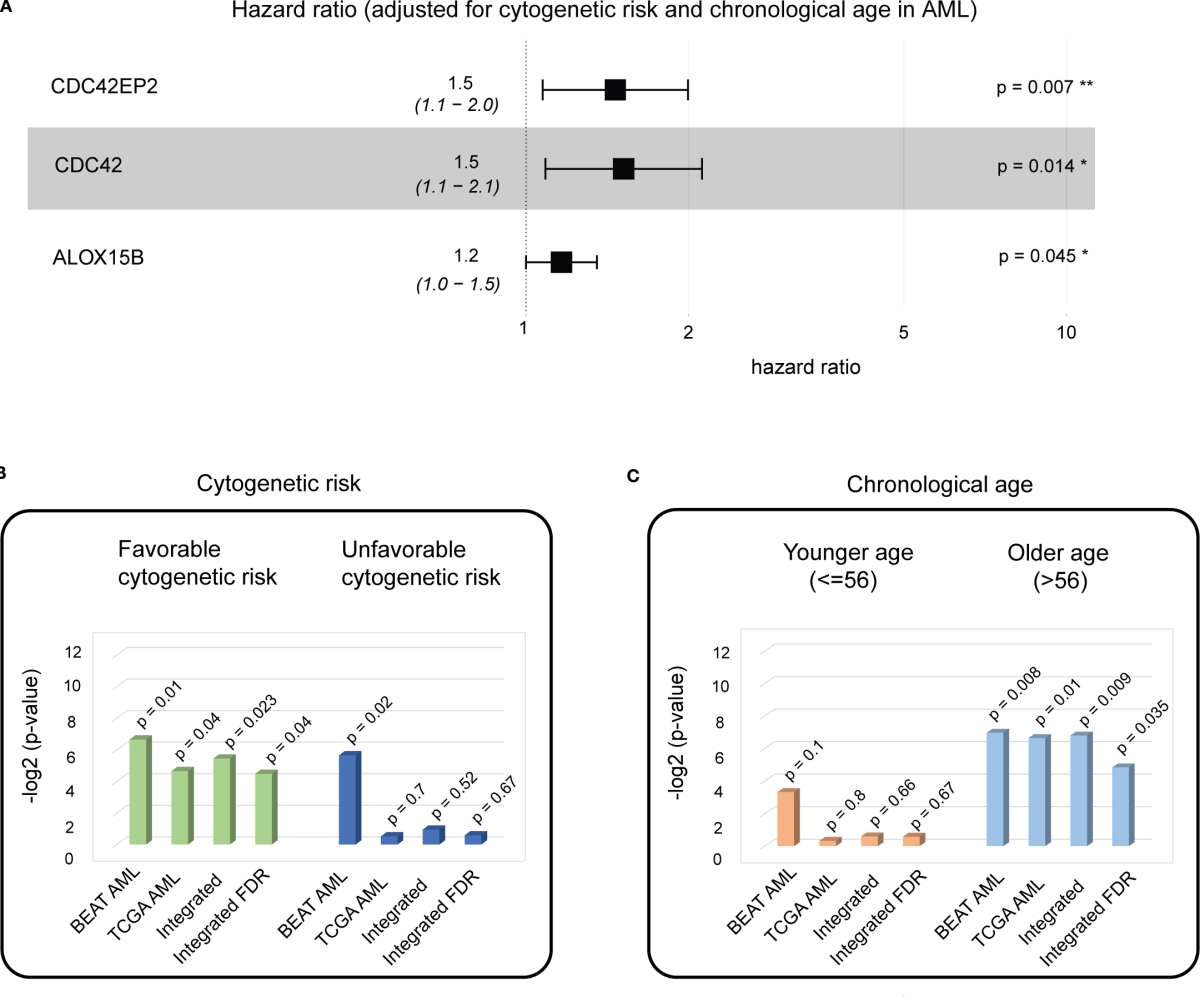
A biological aging analysis of AML cohorts identifies three genes with significant predictive ability in patients with favorable cytogenetic risk scores and of older age. **(A)** A composite hazards plot, where the analysis for each gene was adjusted for cytogenetic risk and chronological age. Central squares indicate the hazard ratio (HR), and whiskers indicate the HR confidence interval. Hazard *p*-values are indicated (^*^ *p*< 0.05; ^**^ *p*< 0.01). **(B)** Cox proportional hazards model analysis for favorable cytogenetic risk patient subgroup (left) and unfavorable cytogenetic risk patient subgroups (right) in the BEAT AML and TCGA AML cohorts. Wald test *p*-values for each dataset and integrated (harmonic mean) *p*-values with and without FDR correction are indicated. **(C)** Cox proportional hazards model analysis for the younger chronological age patient subgroup (left) and the older chronological age patient subgroup (right) in the BEAT AML and TCGA AML cohorts. Wald test *p*-values for each dataset and integrated (harmonic mean) *p*-values with and without FDR correction are indicated.

To test the generalizability of our method, we applied the analysis of biological aging genes to AML, with overall survival as the clinical endpoint. For training purposes, we employed the BEAT AML patient cohort (*n* = 127, see Methods), which comprised primary *de novo* bone marrow samples for patients with AML with a wide chronological age range (2.0–84.0) and the most balanced favorable and unfavorable cytogenetic risk groups (40:60, Methods). To identify AML-specific biological aging genes, we utilized 52 genes as input variables in the univariable Cox proportional hazards model analysis (adjusted for cytogenetic risk and chronological age), with overall survival as a clinical endpoint. This analysis identified three genes with a Cox proportional hazards *p*-value< 0.05 significantly positively associated with AML overall survival (HR > 1, the higher the gene expression the more favorable prognosis the patient will have) (**Figure 5A**, **Supplementary Table S3**, **Supplementary Materials**): CDC42EP2 (Cox proportional hazards model HR = 1.4887, *p*-value = 0.0071), CDC42 (Cox proportional hazards model HR = 1.5161, *p*-value = 0.0144), and ALOX15B (Cox proportional hazards model HR = 1.228, *p*-value = 0.0452). VIF analysis did not identify any multicollinearity among the three genes (see Methods), allowing for a multivariable additive Cox proportional hazards model (adjusted for cytogenetic risk and chronological aging), which demonstrated the significant benefit of evaluating the three genes as one additive model (Wald test *p*-value = 3*e*−06).

Gene ontology and pathway enrichment analyses of the three genes identified RHOQ GTPase Cycle, MAPK6/MAPK4 signaling, and CDC42 GTPase cycle as significant (FDR-corrected chi-square test *p*-value< 0.05). MAPK6/MAPK4 signaling regulates cell morphology, migration, endocytosis, and cell cycle progression, and its dysregulation is a known marker of cancer (24). Interestingly, cell polarity determinant CDC42 controls division symmetry to block leukemia cell differentiation and is involved in AML development (25), while CDC42 GTPase cycle signaling is a known player in aging and age-related diseases (26), nominating these genes as valuable markers for further investigation.

To investigate if a significant association of the three biological aging genes with overall survival is attributed to a specific patient subgroup (that might be driving significance of the association), we have performed stratified Cox proportional hazards model analysis on the patient subgroups stratified by the (1) cytogenetic risk at diagnosis and (2) chronological age, which are known prognostic factors for AML (**Figures 5B, C**).

For stratified analysis based on the cytogenetic risk, the BEAT AML cohort was divided into two patient subgroups: (i) with favorable cytogenetic risk (*n* = 53) and (ii) with unfavorable cytogenetic risk (*n* = 74). Multivariable additive Cox proportional hazards model demonstrated a significant association of the three identified genes with overall survival in patient groups with favorable cytogenetic risk (Cox proportional hazards model Wald test *p*-value = 0.01) and unfavorable cytogenetic risk (Cox proportional hazards model Wald test *p*-value = 0.02).

For stratified analysis based on chronological age, the BEAT AML cohort was divided into two patient subgroups: (i) younger chronological age (≤ 56, *n* = 64) and older age (>56, *n* = 63), with 56 years as the median age in the BEAT AML cohort. Multivariable additive Cox proportional hazards model demonstrated a significant association of the three identified genes with overall survival for the older chronological age group (Cox proportional hazard model Wald test *p*-value = 0.008).

To confirm these findings, we utilized TCGA AML patient cohort (*n* = 151, see Methods), which had a comparable to BEAT AML sample collection protocol (i.e., primary *de novo* bone marrow samples) and clinical endpoint (i.e., overall survival). TCGA AML dataset was similarly stratified on cytogenetic risk into (i) favorable (*n* = 30) and (ii) unfavorable (*n* = 121) subgroups (**Figure 5B**). Similarly to the BEAT AML dataset, chronological age stratification on TCGA AML cohort defined patients with (i) age below or at 56 (*n* = 76) and (ii) age above 56 (*n* = 75) (**Figure 5C**).

Stratified analysis on cytogenetic risk demonstrated a significant association of the three biological aging genes to overall survival in the cytogenetic favorable group (multivariable additive Cox proportional hazards model Wald test *p*-value = 0.04, **Figure 5B**). Stratified analysis on chronological age, demonstrated significant association in the older age (> 56) subgroup (multivariable additive Cox proportional hazards model Wald test *p*-value = 0.01, **Figure 5C**).

To make final conclusions (as in PC analysis), we have further integrated our findings from both cohorts using FDR-corrected harmonic mean (**Figures 5B, C**, **Methods**), which nominated a significant association of the identified genes with overall survival in AML in the favorable cytogenetic risk subgroup (FDR-corrected integrated *p*-value = 0.04) (**Figure 5B**) and the older age (> 56) subgroup (FDR-corrected integrated *p*-value = 0.035) (**Figure 5C**), which builds a foundation for further patient stratification in these patient groups using identified three biological aging genes, allowing for personalized disease management and therapeutic planning in AML patients.

### 3.4 Robustness analysis: comparison to randomly selected genes and other markers of PC and AML progression

To ensure the robustness of our predictions in both PC and AML, we performed (i) a comparison of the predictive ability of our findings to the predictive ability of the equally sized (i.e., four or three, respectively) gene group selected at random; and (ii) a comparison of the predictive ability of our findings to the predictive ability of the known markers of PC and AML progression, as appropriate.

#### 3.4.1 Random modeling

**Figure 6:**
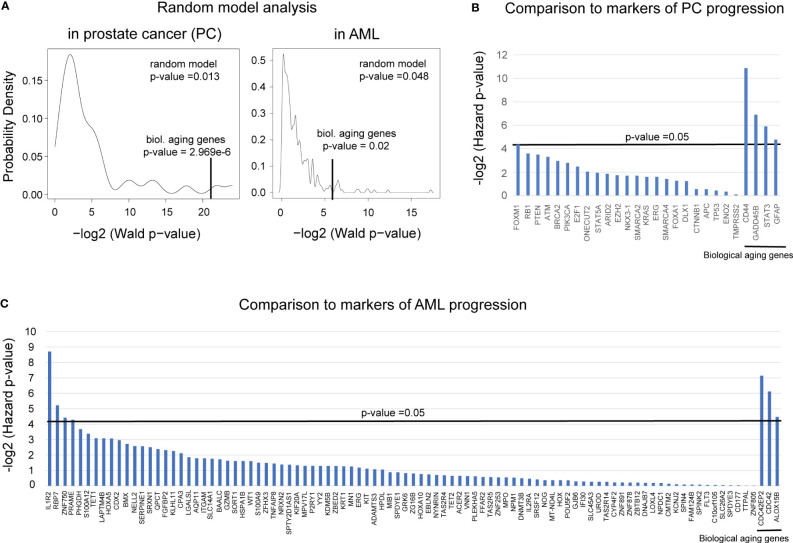
Comparison to randomly selected genes and known markers of progression demonstrated the robustness of PC and AML results. **(A)** A random model plot depicting the distribution of *p*-values for a random set of (left) four genes for PC and (right) three genes for AML ran 10,000 times. *p*-values were estimated using the Wald test from the Cox proportional hazards model (*x*-axis) applied to four or three random genes, respectively. The original Wald test *p*-value for the identified four or three biological aging genes, respectively, is indicated with a vertical black line. **(B)** Plot comparing the predictive ability of the known markers of prostate cancer aggressiveness to predict time to BCR to the predictive ability of the four identified biological aging genes (four most right vertical bars). The predictive ability was evaluated using the Cox proportional hazards model, adjusted for Gleason and chronological age. Hazards *p*-values are indicated. The horizontal black bar indicates a *p*-value threshold of 0.05. **(C)** Plot comparing the predictive ability of the known markers of AML aggressiveness to predict time to overall survival to the predictive ability of the three identified biological aging genes (three most right vertical bars). The predictive ability was evaluated using the Cox proportional hazards model, adjusted for cytogenetic risk and chronological age. Hazard *p*-values are indicated. The horizontal black bar indicates a *p*-value threshold of 0.05.

First, to ensure that the biological aging genes outperform the predictive ability of the genes chosen at random, we built a random model using the Taylor et al. cohort for prostate cancer and the BEAT AML cohort for AML. In such a model, genes (either four or three, respectively) were selected at random from the list of all available genes in each dataset and subjected to Cox proportional hazards model analysis as a group. The Wald *p*-value was reported, and the process was repeated 10,000 times (**Figure 6A**). For prostate cancer, this analysis confirmed a highly nonrandom ability of the identified four biological aging genes to predict prostate cancer progression (**Figure 6A**, left, nominal random model *p*-value = 0.001), and for AML, it confirmed a highly nonrandom ability of the three identified genes to predict AML overall survival (**Figure 6A**, right, nominal random model *p*-value = 0.048).

#### 3.4.2 Comparison to markers of PC and AML progression

In PC analysis, to compare the predictive ability of the identified four biological aging genes to the predictive ability of the known transcriptomic markers of prostate cancer progression (27–39) (**Figure 6B**), we utilized Cox proportional hazards model analysis on the Taylor et al. dataset. Known prostate cancer genes associated with cancer progression were utilized as input variables, adjusted for Gleason score and chronological aging, with time to BCR as a clinical endpoint. The only known PC aggressiveness gene that remained significant after adjusted analysis was FOXM1 (adjusted Cox proportional hazards model *p*-value = 0.048), which was less significant compared to the identified four biological aging genes (**Figure 5B**, right-most genes: CD44 adjusted *p*-value = 0.00054, GADD45B adjusted *p*-value = 0.0084, STAT3 adjusted *p*-value = 0.0167, GFAP adjusted *p*-value = 0.036). Following this result, we investigated the possibility of utilizing FOXM1 [previously identified by our group as a transcriptional regulator of prostate cancer progression (40, 41)] and the four biological aging genes together for enhanced predictive ability using a multivariable Cox proportional hazards model. Interestingly, when combined into a group of five, in the crude multivariable analysis, FOXM1 remained significant (hazard *p*-value = 0.01) but did not significantly reduce the predictive ability of the four biological aging genes, indicating their independent ability to predict prostate cancer progression. Yet, in the adjusted multivariable analysis for the group of five genes, FOXM1 lost its significance, while the biological aging genes remained significant. Furthermore, in the stratified multivariable analysis (i.e., analysis for the group of patients with higher Gleason scores and for the group of patients of younger age), FOXM1 did not show significance for the higher Gleason score (while the four biological aging genes remained significant) but demonstrated significance for the younger patient group, alongside biological aging genes. Taken together, this PC-specific analysis indicates that biological aging genes in PC offer independent predictive evidence compared to known markers of PC progression, especially for patients with higher Gleason scores, and could be effectively utilized alongside FOXM1 for patients of younger age for enhanced predictions of PC progression.

In the AML analysis (**Figure 6C**), we compared the predictive ability of the identified three biological aging genes to the predictive ability of the known markers of AML aggressiveness (42). Several genes have demonstrated significance in the adjusted analysis: IL-1R2 (adjusted Cox proportional hazards model *p*-value = 0.0024), RBP7 (adjusted Cox proportional hazards model *p*-value = 0.0269), and ZNF750 (adjusted Cox proportional hazards model *p*-value = 0.0467). Following this result, we investigated the possibility of utilizing these three genes (IL-1R2, ZNF750, and RBP7) and the three biological aging genes together to enhance their predictive ability using a multivariable Cox proportional hazards model. Interestingly, when utilized together, IL-1R2, ZNF750, and RBP7 showed significant ability to predict overall survival in a younger age group, as opposed to the CDC42EP2, CDC42, and ALOX15B, which showed a stronger predictive ability in older age group, indicating that both gene groups provide independent evidence and predictive ability for different patient populations. Taken together, this AML-specific analysis indicates that biological aging genes in AML offer independent predictive evidence, compared to known markers of AML progression, especially for older patients.

## 4 Discussion

In this work, we have investigated the role of the biological aging genes in prostate cancer progression and identified four prostate cancer-specific genes (CD44, GADD45B, STAT3, and GFAP) having a significant ability to predict time to BCR in prostate cancer patients, especially for patients with a higher Gleason score and for patients of younger chronological age. Our analysis demonstrated the unique independent predictive ability of these genes compared to currently known markers of prostate cancer progression, nominating them as valuable markers of time to BCR in prostate cancer.

CD44 is a multifunctional cell surface adhesion receptor that is expressed in many cancers [including prostate cancer (43)] and promotes the migration and invasion involved in metastases (44, 45). It is a known prostate cancer stem cell marker (46) that has been nominated as a potential therapeutic target (47) and has been shown to play a role in chemo- and radiotherapy response and resistance in prostate cancer (48, 49), nominating CD44 as a valuable marker and potential therapeutic target, requiring further in-depth investigations.

GADD45B is a growth arrest and DNA-damage-inducible beta gene known as a prognostic biomarker in colorectal adenocarcinoma (50, 51) and a facilitator of metastasis in ovarian cancer through epithelial–mesenchymal transition (52). In prostate cancer, it has been demonstrated to be implicated in the mechanism of therapeutic inhibition of prostate cancer (53) and as a therapeutic target in chemotherapy-resistant prostate cancer (54), opening new potential avenues for therapeutic intervention for patients with dysregulation of biological aging mechanisms.

STAT3 is a transcription factor oncogene involved in the IL-6-JAK-STAT3 signaling cascade that mediates gene expression in response to cell stimuli and thus plays a central role in cell growth and apoptosis across multiple cancers (55–57), and their targeting has shown therapeutic benefits (58–60). In prostate cancer, it has been shown to activate stemness and metastatic progression (61–65), be involved in the regulation of tumor microenvironment (66), and has been shown as an effective therapeutic target (67) and sensitizer to radiation therapy (68), nominating it as a central axis for potential therapeutic interventions in biological age-related prostate cancer progression.

GFAP is a glial fibrillary acidic protein that is a promising biomarker and therapeutic target in glioblastoma (69) and other cancers (70, 71); however, its role in prostate cancer has not been fully explored. One of the bioinformatics investigations nominated GFAP as a potential regulator of immune phenotypes in prostate cancer (72), yet its role in prostate cancer progression provides a new exciting avenue for further investigation, especially in combination with GADD45B, STAT3, and GFAP (and, potentially, FOXM1).

Furthermore, we have generalized our methodology and applied our investigations to AML, which identified three AML-specific genes (CDC42EP2, CDC42, and ALOX15B) with significant ability to predict AML overall survival, especially for patients with favorable cytogenetic risk and for patients of older chronological age.

CDC42EP2 belongs to the Rho GTPase family and is a member of the Borg family of CDC42 effector proteins (73). It is identified in gastrointestinal stromal tumors (74) and is a potential prognostic biomarker in nonsmall cell lung cancer survival (75) and in hepatocellular carcinoma (76). However, its role in AML cancer has not been fully explored yet.

CDC42 also belongs to the GTPase family of the Rho subfamily and is a key downstream regulator of multiple cell signaling receptors, including RAS/MAPK and PI3K/Akt. CDC42 is known for its involvement in myeloid and erythroid cell development in mouse models (77). Therapeutic targeting of CDC42 affects the proliferation and survival of multiple myeloma cells (78) and the spread of metastasis in breast cancer models with HER2 and triple-negative subtypes of breast cancer (79). Furthermore, Wnt signaling inhibition affects hematopoietic stem-cell aging via activation of CDC42 (80, 81), all together nominating it as a potential therapeutic intervention in biological age-related AML progression.

ALOX15B belongs to the lipoxygenase family of nonheme iron dioxygenases, which leads to fatty acid hydroperoxide production. ALOX15B is expressed in human monocyte-derived macrophages, and it is believed that the progression of cytogenetically normal AML could be a dysfunction of immune cells in the bone marrow microenvironment (82, 83), directly connecting it to AML progression and nominating it as a possible mechanism of age-related AML therapeutic intervention.

In this work, we focused on transcriptomic markers of biological aging in prostate cancer and AML; however, we understand that biological age is a complex notion and, in addition to changes in gene expression, includes widely explored DNA methylation and telomere length (84–86), among others. Yet, we have not been able to identify prostate cancer or AML datasets with either DNA methylation or telomere length data available that have appropriate sizes and statistical power for training and testing purposes. However, we foresee that such data will be available in the future, and we will utilize these additional sources of information to refine our analysis.

In summary, genome-wide investigations of biological aging demonstrated their high potential to predict cancer progression and cancer-specific overall survival in PC and AML, respectively. We foresee further investigations of the role of biological aging in cancer and the potential utilization of these genes as biomarkers of prostate cancer and AML progression, which could lead to personalized therapeutic planning and open new avenues for novel therapeutic targeting for patients with mechanisms involved in dysregulated biological aging.

## Data availability statement

The original contributions presented in the study are included in the article/**Supplementary Material**. Further inquiries can be directed to the corresponding author.

## Author contributions

AR downloaded PC data from cBioPortal, processed and performed analysis on PC data, and wrote PC sections of the manuscript. ID downloaded AML data from dbGap and GDC portals, processed and analyzed AML data, and wrote AML sections of the manuscript. SP downloaded PC data from dbGap, processed and performed data analysis for PC datasets and advised on statistical analysis for the manuscript. HP performed a literature search to identify 52 biological aging genes. YL performed random model analysis. MC analyzed pathway enrichment data and contributed to the literature review. CC performed a literature search on biological aging genes. GJ-M ran pathway enrichment analysis and performed a literature review for PC genes. A-RO ran pathway enrichment analysis and performed a literature review for AML genes. IK contributed to the discussion section and advised on the clinical significance of the identified biological aging genes. AM conceived and wrote the manuscript. All authors contributed to the article and approved the submitted version.
